# Robustness of inferences in risk and time experiments to lifecycle asset integration

**DOI:** 10.1371/journal.pone.0332888

**Published:** 2025-09-29

**Authors:** AJ A. Bostian, Christoph Heinzel

**Affiliations:** 1 School of Social Sciences and Humanities, University of Tampere, Tampere, Finland; 2 INRAE UMR SMART, Institut Agro, Rennes, France; University of Pretoria, SOUTH AFRICA

## Abstract

Experiment participants can engage in unobservable asset integration, mentally incorporating non-experimental “field” resources into an ostensibly controlled scenario. We extend asset integration to include lifecycle intertemporal tradeoffs. Our model shows that interference from purely exogenous field resources can be controlled with a simple wealth-level adjustment. Highly substitutable endogenous resources, by contrast, require modeling the entire experiment-field interaction. We examine the practical implications using three classic experiments on risk and time. As interference worsens, experimental decisions tend to exhibit an attenuation toward less risk aversion and more patience. This occurs reliably with household-scale resources, but pocket-change amounts can also cause problems.

## 1 Introduction

Experiments that elicit risk and time preferences are susceptible to asset integration, a design bypass that occurs when participants mentally incorporate their own financial resources into a supposedly controlled scenario. Because asset integration is triggered by the scenario itself, its impacts cannot be neutralized with randomization. As an unobservable mental process, it can introduce a particularly unfortunate confound into post hoc utility assessment.

Though it was first discussed in a static context [1], asset integration can appear in dynamic settings just as easily. But, its current adaptation to time is rudimentary: the non-experimental or “field” environment consists merely of recurring static resources. If this is the correct interpretation of asset integration, then the confound can be eliminated rather easily by including those resource levels in the utility argument.

However, participants almost surely do not regard their own field environments as mere successions of resources that they must take as given. Instead, they sew up those field resources into a coherent lifecycle plan. That process taps intertemporal tradeoffs that do not emerge under the recurring-static view. A well-known example is consumption smoothing, which distributes temporally-unbalanced resources more evenly across time. We use the term “lifecycle asset integration” to denote the complications that arise when an experiment is confounded by such factors.

In this paper, we develop a model of lifecycle asset integration, and examine its implications for experiments that investigate risk and time preferences. This model is rooted in the two-period consumption-saving framework, the canonical theory of lifecycle decision making under risk [2–8]. Our extension merges an experimenter’s controlled experimental incentives with a participant’s existing field environment, yielding a joint optimization problem of experimental and field smoothing. This setup lets us pull in several fundamental results from intertemporal choice theory, whose effects on asset integration have not been previously considered.

Our main theoretical result highlights a key difference in the way exogenous and endogenous field resources affect an experiment. Exogenous resources are much like the recurring-static conception of the field. They have no associated smoothing instrument (such as saving), and so cannot be moved across time. Endogenous resources, on the other hand, do involve a smoothing instrument, and thus can be moved across time. Endogenous field instruments germane to this context include participants’ credit cards and bank accounts.

An experiment has no effect whatsoever on exogenous field resources. As a consequence, the appropriate post hoc correction for exogenous field resources is the relatively minor procedure of controlling for their levels in the utility argument [9]. But, this simple control strategy is inadequate for endogenous field resources, because experimental and field smoothing instruments can substitute for each other.

Indeed, our model suggests that the marginal rate of substitution (MRS) between experimental and field smoothing will likely be near 1 in most settings, implying near-perfect substitution. In other words, the existence of the experiment will actually alter the amount of field smoothing, a different component of the utility argument. Simply conditioning on pre-existing field outcomes is insufficient in this case, because the experiment itself will change those outcomes. The appropriate correction here is much more onerous: modeling the entire experiment-field interaction, including the MRS.

To illustrate the practical effects of lifecycle asset integration, we numerically investigate our model’s predictions for three classic laboratory experiments: the risk aversion task of Holt and Laury [10] (HL), and the discounting tasks of Andersen *et al*. [11] (AHLR) and Andreoni and Sprenger [12] (AS). By making simple notational changes to the originals, we show that all three are special cases of our model. We can thus rely on our model from the start to treat each experiment on its own terms first, and then add different integration assumptions.

The goal of these exercises is to examine how experimental predictions change as integration assumptions change, *holding preferences constant*. If those predictions differ substantively, the experiment is not robust to lifecycle asset integration. The “controlled” experimental observations can be readily contaminated by field interactions.

Because the dividing line between experimental and field incentives is very clear with laboratory experiments, those designs are easy to adapt to our model. Notably, asset integration does not spark uniform concern within the laboratory literature. At one extreme, its relevance to static choice-bracketing experiments has been debated vigorously [13,14]. At the other, many temporal experiments simply assume asset integration from the start [11,12,15–17]. Critically, all experimental gains–even the gains from static tasks–are spent within participants’ lifecycle plans, making every experiment potentially vulnerable to lifecycle asset integration.

Our first example, the HL task, requires participants to choose between pairs of safe and risky lotteries. As a static decision, HL does not have its own smoothing instrument, and so it evades the substitution problem between field and experimental smoothing. It is still exposed to the other issues.

Omitting exogenous field resources situates post hoc analysis at the wrong background level. HL assumes this is $0, but we find a strong sensitivity to that assumption. Adding just $0.20 of exogenous field resources causes HL decisions to creep toward risk neutrality, even though preferences are calibrated risk averse. Increasing that level to just $7 makes those decisions completely indistinguishable from risk neutrality.

Omitting endogenous field resources ignores how the task activates the participant’s consumption-smoothing and precautionary motives, two new behaviors introduced by our intertemporal framework. From the perspective of a participant’s lifecycle plan, HL payoffs are a risky windfall that will trigger both. Each one will also push HL decisions toward seemingly neutral interpretations.

Intertemporal choice theory makes straightforward predictions about the operation of endogenous field resources under both motives. In general, the consumption-smoothing motive responds to any change in the mean consumption path by making it less lumpy over time. So, when a new experiment is dropped into a participant’s the lifecycle path, this attitude will prompt the participant to use field instruments to smooth out the new lump. Analogously, the precautionary motive responds to any higher-moment changes in the risk profile. When a new experiment carrying risk is dropped into a participant’s lifecycle path, this attitude will prompt the participant to use field instruments to support the period with the new risk. To reiterate, these are vanilla effects from intertemporal choice theory–our question is how strongly they affect risk and time experiments.

The AHLR task requires participants to choose between pairs of current and future payoffs. Even though AHLR does not explicitly call its decision “saving,” we show that its decision problem nevertheless contains a latent variable corresponding to our model’s experimental smoothing variable. AHLR is exposed to all HL issues, but the omission of endogenous field resources causes two more complications.

First, as with HL, a participant can potentially smooth an AHLR windfall using a field instrument that the experimenter cannot observe. But, because the ALHR decision is itself a smoothing decision, the participant can also satisfy that smoothing desire during the experiment using the observable instrument. This fact underscores the bigger problem: participants now have two *highly substitutable* smoothing instruments at their disposal.

Given that, a participant will try to save as much as possible with the instrument that provides the better outcome. The meaning of “better” depends on the field consumption path. If that path already has high future consumption, for instance, even large experimental returns may not induce any saving. These predictions are quite different from the ones that emerge when assuming the experimental instrument operates in isolation in the laboratory.

The AS task can be viewed as an extension of AHLR that allows participants to choose their own smoothing amounts. That freedom of choice clearly calls out the near-unit MRS between experimental and field smoothing. In fact, it is quite easy to drive experimental saving to its boundary values by making the experimental incentives too stingy or too rich relative to the field. Consistent with that prediction, Andreoni and Sprenger do observe an unusually large fraction of boundary decisions.

HL, AHLR, and AS each estimate structural parameters assuming expected utility (EU). Our model uses recursive utility (RU) instead [8,18]. The reason is flexibility: RU allows smoothing preferences to be uncoupled from risk preferences, while EU fuses them. But, because EU is a special case of RU, our framework still covers all EU analysis. To be clear, our results do not hinge on whether a participant is an EU or RU decision maker. Rather, when asset integration has a *lifecycle* nature, it is easier to illustrate the interference pattern from the stance that relative risk aversion (RRA) and the elasticity of intertemporal substitution (EIS) are not bound functionally. That allows us to calibrate each domain with plausible values from its own literature.

Prior research hints at aspects of our results. Cubitt and Read [16] discuss interference between temporal experimental choices and field variables under EU, but risk does not enter that assessment. Schechter [19] performs a similar evaluation, calibrating an intertemporal utility function with the results of a static risk task. Coble and Lusk [20] examine AHLR with isoelastic RU preferences, as do Miao and Zhong [21] when examining AS. Both find empirical support for RU over EU, but neither include asset integration. In a large Danish cohort, Epper *et al*. [22] find that patience in AS positively correlates with wealth across the lifecycle. Below, we gather all these existing matters regarding the experiment-field interaction, along with some new ones, into a single analytical framework.

## 2 Lifecycle asset integration

To formally capture the interaction between an experimental stimulus and a participant’s lifecycle path, we extend a two-period consumption-saving model. This extension differentiates field incentives from experimental ones. To that end, the field elements of this model are denoted with *f* superscripts, and the experimental elements with *e* superscripts. Risky variables are denoted with tildes. Risk occurs only in period 2 by convention. Field and experimental risks are considered independent by construction.

The field incentives are the period 1 income $y_{1}^{f}$, the period 2 exogenous risky income ${\tilde{y}}_{2}^{f}$, and the period 2 gross return to saving $R_{2}^{f}$ (net return $r_{2}^{f}$). The analogous experimental incentives are $y_{1}^{e}$, ${\tilde{y}}_{2}^{e}$, and $R_{2}^{e}$. The field incentives are outside the experimenter’s control, while the experimental incentives are the experimenter’s manipulation.

The participant chooses field saving $s_{1}^{f}$ and experimental saving $s_{1}^{e}$ to maximize the RU objective over lifecycle consumption $c_{1}^{f},{\tilde{c}}_{2}^{f}$:

$$\max_{s_{1}^{f},s_{1}^{e}}\;u\left(c_{1}^{f}\right)+\beta u\left(CE\left({\tilde{c}}_{2}^{f}\right)\right)\;\text{s.t.}\;\left\{ \begin{array}{c} y_{1}^{f}+y_{1}^{e}=c_{1}^{f}+s_{1}^{f}+s_{1}^{e}\\ {\tilde{y}}_{2}^{f}+{\tilde{y}}_{2}^{e}+s_{1}^{f}R_{2}^{f}+s_{1}^{e}R_{2}^{e}={\tilde{c}}_{2}^{f}\\ -{\tilde{y}}_{2}^{f}\leq s_{1}^{f}\leq y_{1}^{f}\\ 0\leq s_{1}^{e}\leq y_{1}^{e} \end{array}\right.$$ (1)

We call $s_{1}^{f}$ and $s_{1}^{e}$ “saving,” as does most of the literature. That terminology certainly conveys $s_{1}^{f}$’s and $s_{1}^{e}$’s function in (1). But, more precisely, those variables reflect a generic two-channel smoothing framework that allocates consumption across time. This nuance allows us to bring experiments under the umbrella of (1) that do not explicitly invoke the language of saving, but nevertheless set up a latent smoothing instrument that behaves like $s_{1}^{e}$. Indeed, only a few experiments explicitly style their tasks as “saving” [23–26].

RU’s notion of time preference has two parts: the utility discount factor *β*, and the intertemporal felicity function *u* that controls consumption smoothing. This is an important distinction from EU, where time preference is considered to be *β* alone (or some variant). The risk preference *ψ* determines the certainty equivalent of future consumption


$$CE\left({\tilde{c}}_{2}^{f}\right)\equiv \psi^{-1}\left(E_{1}^{f}E_{1}^{e}\left[\psi \left({\tilde{c}}_{2}^{f}\right)\right]\right)$$

Unlike *u*, *ψ* is an EU function. RU thus contains a utility-of-wealth function *ψ*, and a utility-of-consumption function *u*. RU collapses to EU if u=ψ [27].

In lifecycle models like this one, nearly any incentive–field or experimental–will activate *β*, *u*, and *ψ* simultaneously [28]. That is equally true of risk, somewhat counterintuitively [8]. The lifecycle risk response differs from the more familiar static response in two other ways. First, a risk’s *n*^*th*^ moment activates the $n\;+\;1^{th}$ utility derivative, not the *n*^*th*^ [7]. The lifecycle response thus depends on *ψ* derivatives *higher* than $\psi^{\prime \prime }$ [18]. By corollary, the well-known Arrow-Pratt coefficient $-\psi^{\prime \prime }/\psi^{\prime }$ has nothing to say about a participant’s reaction to lifecycle risk.

The first two constraints in (1) show how experimental and field assets get integrated into the participant’s lifecycle plan. The vehicle is lifecycle consumption. Both period 1 consumption $c_{1}^{f}=\left(y_{1}^{f}+y_{1}^{e}\right)-\left(s_{1}^{f}+s_{1}^{e}\right)$ and period 2 consumption ${\tilde{c}}_{2}^{f}=\left({\tilde{y}}_{2}^{f}+{\tilde{y}}_{2}^{e}\right)+\left(s_{1}^{f}R_{2}^{f}+s_{1}^{e}R_{2}^{e}\right)$ contain an amalgam of experimental and field resources.

The properties of *u* and *ψ* that guarantee a unique maximum for (1) also guarantee interior equilibrium consumption levels $c_{1}^{f*}$ and ${\tilde{c}}_{2}^{f*}$. For that reason, we simply assert the equality of the first two constraints. But, the fact that the consumption path is interior does not mean the saving decisions are as well. Absent additional information on preferences and incentives, the constraints on $s_{1}^{f*}$ and $s_{1}^{e*}$ must stay inequalities.

Field saving $s_{1}^{f}$ can be positive or negative. We place a loose restriction on saving and borrowing via this channel: the participant’s own lifecycle field income. We place a much stronger restriction on experimental saving $s_{1}^{e}$: the participant cannot borrow at all during the experiment, or save more than $y_{1}^{e}$. This reflects a common design constraint. Ethical considerations usually prohibit participants from investing–and potentially losing–their own field resources in an experiment. Their decisions must always fit within the resource levels endowed by the experimenter.

The interpretation of field resources $y_{1}^{f}$ and ${\tilde{y}}_{2}^{f}$ is context-specific. As a rule, those terms express incentives that the participant *perceives* to be relevant to the experimental decision $s_{1}^{e}$, but are not actually part of the experiment. To reiterate, these perceptions are not entirely under the experimenter’s control, nor are they fully observable. Thus, $y_{1}^{f}$ and ${\tilde{y}}_{2}^{f}$ should not be read merely as attributes that the experimenter could account for in principle. They can also reflect traits the experimenter has no hope of observing.

Even though the experimental incentives are “controlled” in the sense of being exogenous, the participant’s experimental and field decisions are tightly coupled. The nature of that coupling can be seen by writing the 1st order conditions in Euler form:

$$s_{1}^{f}:\;E_{1}^{f}E_{1}^{e}\left[\beta \cdot \left(\frac{u^{\prime }\left(CE\left({\tilde{c}}_{2}^{f}\right)\right)}{u^{\prime }\left(c_{1}^{f}\right)}/\frac{\psi^{\prime }\left(CE\left({\tilde{c}}_{2}^{f}\right)\right)}{\psi^{\prime }\left({\tilde{c}}_{2}^{f}\right)}\right)\cdot R_{2}^{f}\right]-1⋚0$$ (2a)

$$s_{1}^{e}:\;E_{1}^{f}E_{1}^{e}\left[\beta \cdot \left(\frac{u^{\prime }\left(CE\left({\tilde{c}}_{2}^{f}\right)\right)}{u^{\prime }\left(c_{1}^{f}\right)}/\frac{\psi^{\prime }\left(CE\left({\tilde{c}}_{2}^{f}\right)\right)}{\psi^{\prime }\left({\tilde{c}}_{2}^{f}\right)}\right)\cdot R_{2}^{e}\right]-1⋚0$$ (2b)

The inequalities follow from the fact that $s_{1}^{f*}$ and $s_{1}^{e*}$ are not necessarily interior. It is important to note that the first equation will apply even if the experimental task is static (i.e., if $s_{1}^{e}\equiv 0$). Static manipulations can still alter $c_{1}^{f}$ and ${\tilde{c}}_{2}^{f}$, and hence $s_{1}^{f}$.

Individually, (2a) and (2b) are examples of a discounted-return equilibrium condition $E_{t}\left(m_{t+1}R_{t+1}\right)⋚1$ that arises ubiquitously in dynamic models [29]. Like that generic condition, the left sides here take the form of discounted expected returns. The quantity corresponding to the discount function *m*_*t* + 1_ is the participant’s stochastic discount factor (SDF)

$$\beta \cdot \left(\frac{u^{\prime }\left(CE\left({\tilde{c}}_{2}^{f}\right)\right)}{u^{\prime }\left(c_{1}^{f}\right)}/\frac{\psi^{\prime }\left(CE\left({\tilde{c}}_{2}^{f}\right)\right)}{\psi^{\prime }\left({\tilde{c}}_{2}^{f}\right)}\right)$$ (3)

As a system, (2a) and (2b) are akin to the 1st order conditions of a multi-asset portfolio model [30]. A key feature of that setting is that all allocations are pinned down by the same *m*_*t* + 1_. Our model shares this characteristic, with the same SDF pinning down both $s_{1}^{f}$ and $s_{1}^{e}$. As with the multi-asset model, that fact carries an important equilibrium implication: a change in one saving amount will alter the SDF, and thereby affect the other’s discounted return–and hence change the other saving amount too.

In light of that, the SDF in (3) is best described as the behavioral transmission mechanism between the experiment and field. This transmission is regulated by *β* and two marginal rates of substitution. The one involving *u* captures the consumption-smoothing implications of the joint decision, while the one involving *ψ* captures the risk-aversion implications.

Moreover, the fact that the SDF contains all three behavioral primitives carries an important implication for interpreting the experimental outcome $s_{1}^{e}$. Namely, the presence of lifecycle asset integration will cause *all preference dimensions*–consumption smoothing, risk aversion, and discounting–to activate simultaneously in response to any set of incentives. Hence, even if the experimenter’s intent is to design a manipulation $\left\{y_{1}^{e},{\tilde{y}}_{2}^{e},R_{2}^{e}\right\}$ that activates only risk attitudes, or only smoothing attitudes, or only the pure rate of time preference, lifecycle asset integration will nevertheless activate everything.

The elasticity of substitution between experimental and field decisions makes the consequences of SDF transmission unmistakable:

$$\epsilon^{e,f}=\frac{ds_{1}^{f}}{ds_{1}^{e}}\cdot \frac{s_{1}^{e}}{s_{1}^{f}}\;=-\frac{u^{\prime \prime }\left(c_{1}^{f}\right)+\beta \left[u^{\prime \prime }\left({\tilde{c}}_{2}^{f}\right)CE^{\prime }{\left({\tilde{c}}_{2}^{f}\right)}^{2}+u^{\prime }\left({\tilde{c}}_{2}^{f}\right)CE^{\prime \prime }\left({\tilde{c}}_{2}^{f}\right)\right]\cdot \frac{1}{2}\left(R_{2}^{f}+R_{2}^{e}\right)R_{2}^{e}}{u^{\prime \prime }\left(c_{1}^{f}\right)+\beta \left[u^{\prime \prime }\left({\tilde{c}}_{2}^{f}\right)CE^{\prime }{\left({\tilde{c}}_{2}^{f}\right)}^{2}+u^{\prime }\left({\tilde{c}}_{2}^{f}\right)CE^{\prime \prime }\left({\tilde{c}}_{2}^{f}\right)\right]\cdot \frac{1}{2}\left(R_{2}^{f}+R_{2}^{e}\right)R_{2}^{f}}\cdot \frac{s_{1}^{e}}{s_{1}^{f}}$$ (4)

S1 Appendix contains the derivation of $\epsilon^{e,f}$. The MRS part $\left|ds_{1}^{f}/ds_{1}^{e}\right|$ describes how well the participant’s field saving can replace experimental saving, and vice versa. Ideally, this quantity would be 0. Experimental and field decisions would not affect each other at all in that case, signaling that the experimenter’s manipulation remains confined to the experiment.

But, the MRS’s numerator and denominator differ by only their last terms, $R_{2}^{e}$ and $R_{2}^{f}$. Thus, barring any implausibly extreme experimental incentives, the MRS is likely to be close to 1. This unfortunately means that experimental smoothing and field smoothing can perfectly substitute for each other. The experimenter must then worry about contamination from not only the exogenous sources $y_{1}^{f}$ and ${\tilde{y}}_{2}^{f}$, but also the endogenous source $s_{1}^{f}R_{2}^{f}$.

Because many experimental studies assume EU, it is worth noting the EU SDF’s behavior when it is viewed as an RU special case. As a rule, EU requires the “reduction of compound lotteries” axiom to hold in all circumstances. In lifecycle settings, this means it must hold within and across time. RU loosens that into “temporal consistency,” which requires conformity within time only [27,31]. Temporal consistency materializes in SDF (3) as the distinction between risk and intertemporal substitution.

That distinction becomes irrelevant under EU. In the EU case u=ψ, the SDF collapses all the way to


$$\beta \cdot \frac{u^{\prime }\left({\tilde{c}}_{2}^{f}\right)}{u^{\prime }\left(c_{1}^{f}\right)}=\beta \cdot \frac{\psi^{\prime }\left({\tilde{c}}_{2}^{f}\right)}{\psi^{\prime }\left(c_{1}^{f}\right)}$$

Notably, in this situation, it does not matter whether the felicity function is taken to be an “intertemporal preference” *u* or a “risk preference” *ψ*. The same decisions will be made under either interpretation.

That result, if valid, provides a powerful design shortcut. Namely, the experimenter can elicit a participant’s risk preference and immediately treat it as the intertemporal preference, or vice versa. However, a good deal of empirical literature, particularly from macroeconomics, rejects the hypothesis that risk substitution and intertemporal substitution have equal elasticities [32–34].

Before calibrating our model to the three example designs, we note two important ways that we have narrowed focus relative to the RU literature. First, this two-period framework permits only two temporal actions: smoothing forward in time, or backward in time. A multiperiod value function would certainly give a more nuanced time path. But, that model generates essentially the same 1st order conditions as ours, while greatly increasing the difficulty of the numerical exercises (policy functions instead of scalars). Worse, we could not appeal to the theoretical corpus for any intuition, as that literature largely operates with two periods. Because we are not concerned with the time path of field smoothing per se, but with whether that smoothing interferes with experimental decisions, we stick with this simpler “forward or backward” approach.

Second, limiting risk to the exogenous channels ${\tilde{y}}_{2}^{f}$ and ${\tilde{y}}_{2}^{e}$ greatly simplifies our discussion of risk attitudes and risk responses. When risk exposure is endogenous with a choice variable, the lifecycle risk response gets tortuous [35]. Risks on ${\tilde{R}}_{2}^{f}$ and ${\tilde{R}}_{2}^{e}$ are prime examples of endogenous exposure, because the amounts at risk $s_{1}^{f}{\tilde{R}}_{2}^{f}$ and $s_{1}^{e}{\tilde{R}}_{2}^{e}$ then depend on the choice variables $s_{1}^{f}$ and $s_{1}^{e}$. None of our three examples include return risk, and so we do not lose much intuition by narrowing our focus in this way.

## 3 Lifecycle asset integration in three experiments

### 3.1 Holt and Laury

As a static experiment, HL has no internal concept of time. Even so, we will motivate it with time notation consistent with (1). Within the context of HL itself, that notation is pure surplusage: nothing would be gained or lost by adding or removing it. But, including it from the outset makes the transition to (1) much easier.

Consistent with the conventions noted above, we label the HL risk “period 2.” Of course, period 1 is usually considered the “present” and period 2 the “future,” and so we might more naturally place that risk in period 1. But, that would require either breaking the timing convention, or adding more periods. The former would obfuscate otherwise crisp theoretical predictions about the roles of consumption-smoothing and precautionary motives, and the latter would yield more convoluted comparison equations. Neither is necessary to understand the intuition on HL interference from lifecycle factors. To the extent there is an ideal way to frame the timing, the fact that our version ultimately reduces to HL after removing all field resources, indicates it respects both HL and the goals of this exercise.

Table 1 presents the baseline HL task, a multiple price list (MPL) of ten safe and risky lotteries. The payoffs for each safe lottery ${\tilde{y}}_{2}^{e,safe}$ and each risky lottery ${\tilde{y}}_{2}^{e,risky}$ are fixed throughout, and the payoffs of ${\tilde{y}}_{2}^{e,risky}$ always entail more spread. Moving down the MPL, the probabilities increasingly favor the higher payoffs. The safe and risky lotteries have equal means at line 5. The safe lottery’s mean is higher above line 5, and the risky lottery’s mean is higher below it.

**Table 1:** Holt and Laury’s baseline MPL.

Line	${\tilde{y}}_{2}^{e,safe}$ ($)		${\tilde{y}}_{2}^{e,risky}$ ($)	
1	1/10 of 2.00	9/10 of 1.60	1/10 of 3.85	9/10 of 0.10
2	2/10 of 2.00	8/10 of 1.60	2/10 of 3.85	8/10 of 0.10
3	3/10 of 2.00	7/10 of 1.60	3/10 of 3.85	7/10 of 0.10
4	4/10 of 2.00	6/10 of 1.60	4/10 of 3.85	6/10 of 0.10
5	5/10 of 2.00	5/10 of 1.60	5/10 of 3.85	5/10 of 0.10
6	6/10 of 2.00	4/10 of 1.60	6/10 of 3.85	4/10 of 0.10
7	7/10 of 2.00	3/10 of 1.60	7/10 of 3.85	3/10 of 0.10
8	8/10 of 2.00	2/10 of 1.60	8/10 of 3.85	2/10 of 0.10
9	9/10 of 2.00	1/10 of 1.60	9/10 of 3.85	1/10 of 0.10
10	10/10 of 2.00	0/10 of 1.60	10/10 of 3.85	0/10 of 0.10

On each line, the participant indicates a preference for the safe or risky lottery. That decision is governed by the comparison

$$E_{1}^{e}\left[\psi \left({\tilde{y}}_{2}^{e,safe}\right)\right]⋛E_{1}^{e}\left[\psi \left({\tilde{y}}_{2}^{e,risky}\right)\right]$$ (5)

Given the MPL’s ordering, a risk-neutral participant would switch from safe to risky at line 5. A risk-averse participant would switch further down. HL also includes decision error by layering a discrete-choice framework on top of (5). To make our interference implications as crisp as possible, we will abstract from these errors.

Table 2 summarizes how these incentives translate to (1). Two items are of particular note. First, because HL does not have a smoothing instrument, the $s_{1}^{e}$ aspect does not apply. Second, HL does not address the field at all.

**Table 2:** Translation between model (1) and HL.

Lifecycle Variable	HL “Safe”	HL “Risky”
$y_{1}^{e}$	0	0
$s_{1}^{e}$	−	−
${\tilde{y}}_{2}^{e}$	${\tilde{y}}_{2}^{e,safe}$	${\tilde{y}}_{2}^{e,risky}$
$R_{2}^{e}$	−	−
$y_{1}^{f}$	−	−
${\tilde{y}}_{2}^{f}$	−	−
$s_{1}^{f}$	−	−
$R_{2}^{f}$	−	−

The comparison analogous to (5) under lifecycle asset integration is


$$u\left(c_{1}^{f,safe}\right)+\beta u\left(CE\left({\tilde{c}}_{2}^{f,safe}\right)\right)⋛u\left(c_{1}^{f,risky}\right)+\beta u\left(CE\left({\tilde{c}}_{2}^{f,risky}\right)\right)$$

Applying the values in Table 2 and expanding terms yields

$$u\left(y_{1}^{f}-s_{1}^{f,safe}\right)+\beta u\left(\psi^{-1}\left(E_{1}^{f}E_{1}^{e,safe}\left[\psi \left({\tilde{y}}_{2}^{f}+{\tilde{y}}_{2}^{e,safe}+s_{1}^{f,safe}R_{2}^{f}\right)\right]\right)\right)⋛\;u\left(y_{1}^{f}-s_{1}^{f,risky}\right)+\beta u\left(\psi^{-1}\left(E_{1}^{f}E_{1}^{e,risky}\left[\psi \left({\tilde{y}}_{2}^{f}+{\tilde{y}}_{2}^{e,risky}+s_{1}^{f,risky}R_{2}^{f}\right)\right]\right)\right)$$ (6)

Critically, the participant’s field incentives $\left\{y_{1}^{f},{\tilde{y}}_{2}^{f},R_{2}^{f}\right\}$ do not change on either side of this comparison. The question is whether the participant’s field decision $s_{1}^{f}$ does.

If $s_{1}^{f}$ does not actually change, then the lifecycle comparison (6) collapses right back to HL’s static one. To see this, note that setting $s_{1}^{f,safe}=s_{1}^{f,risky}$ lets (6) simplify to


$$E_{1}^{f}E_{1}^{e,safe}\left[\psi \left({\tilde{y}}_{2}^{e,safe}+\left({\tilde{y}}_{2}^{f}+s_{1}^{f}R_{2}^{f}\right)\right)\right]⋛E_{1}^{f}E_{1}^{e,risky}\left[\psi \left({\tilde{y}}_{2}^{e,risky}+\left({\tilde{y}}_{2}^{f}+s_{1}^{f}R_{2}^{f}\right)\right)\right]$$

This is just (5) with an additional field term ${\tilde{\omega }}_{2}^{f}={\tilde{y}}_{2}^{f}\;+\;s_{1}^{f}R_{2}^{f}$ in the utility argument. So, if $s_{1}^{f}$ truly does not change, the only essential refinement to HL is to condition on those field assets and risks [9,36,37].

However, it is more likely that each side of (6) will yield different values of $s_{1}^{f}$. The reason is that ${\tilde{c}}_{2}^{f}$ has different means and variances on each side:


$$E_{1}\left({\tilde{c}}_{2}^{f}\right)=E_{1}^{f}\left({\tilde{y}}_{2}^{f}\right)+E_{1}^{e}\left({\tilde{y}}_{2}^{e}\right)+s_{1}^{f}R_{2}^{f}$$

$$V_{1}\left({\tilde{c}}_{2}^{f}\right)=V_{1}^{f}\left({\tilde{y}}_{2}^{f}\right)+V_{1}^{e}\left({\tilde{y}}_{2}^{e}\right)$$

These two moments influence the lifecycle in well-known ways [6,7,18]. The most consequential response is the one to $E_{1}\left({\tilde{c}}_{2}^{f}\right)$, which triggers consumption smoothing. Second-most consequential is the one to $V_{1}\left({\tilde{c}}_{2}^{f}\right)$, which triggers precaution. All of ${\tilde{c}}_{2}^{f}$’s higher moments trigger precaution as well, but their contributions are negligible here.

Because $E_{1}^{e,safe}\left({\tilde{y}}_{2}^{e,safe}\right)⋚E_{1}^{e,risky}\left({\tilde{y}}_{2}^{e,risky}\right)$, the field saving amounts $s_{1}^{f,safe}$ and $s_{1}^{f,risky}$ will generally have different consumption-smoothing components. The lottery with the higher mean will induce more smoothing. Because risk is dated period 2, that lottery will have lower $s_{1}^{f}$, and thus higher $c_{1}^{f}$.

Similarly, because $V_{1}^{e,safe}\left({\tilde{y}}_{2}^{e,safe}\right)<V_{1}^{e,risky}\left({\tilde{y}}_{2}^{e,risky}\right)$, the field saving amounts $s_{1}^{f,safe}$ and $s_{1}^{f,risky}$ will have different precautionary components as well. The risky lottery will induce more precaution for sure. Because risk is dated period 2, the risky lottery will have higher $s_{1}^{f,risky}$, and thus lower $c_{1}^{f,risky}$.

Comparing (5) and (6) highlights two specification errors that will arise during structural estimation by failing to include lifecycle asset integration. The root of both is the omitted field term ${\tilde{\omega }}_{2}^{f}={\tilde{y}}_{2}^{f}+s_{1}^{f}R_{2}^{f}$. The first is failing to control for the exogenous field resources ${\tilde{y}}_{2}^{f}$. The error here arises from implicitly assuming ${\tilde{y}}_{2}^{f}=0$, thereby situating the participant’s decisions at the wrong field level. Because HL’s *ψ* allows increasing, decreasing, and constant RRA, centering the decision at the correct ${\tilde{y}}_{2}^{f}$ level is of paramount concern.

The second is failing to account for any endogenous field resources $s_{1}^{f}R_{2}^{f}$. This also injects a background-level problem, but it is further compounded by implicitly assuming that the participant’s field saving remains the same (at $s_{1}^{f}=0$) on both sides of (6). This treats an endogenous field resource as if it is exogenous.

Casting endogenous resources as exogenous fails to appreciate the full array of preferences operating on ${\tilde{c}}_{2}^{f}$. The specific problem is that the $s_{1}^{f}R_{2}^{f}$ component is governed by *u*, not just *ψ*. A structural model like (5) with only *ψ* would improperly assign all of the experimental decision to risk attitudes, when part is actually caused by smoothing attitudes. Such “*ψ* estimates” would be an uninterpretable mash of risk and intertemporal preferences.

Without question, the second issue requires a much more invasive correction than the first. The solution to omitting exogenous field resources is the usual prescription for omitted-variable bias: include those resources. That correction does not require changing the underlying structural model (5). The solution to omitting endogenous field resources, on the other hand, requires specifying how those resources interact with the experiment. Because that interaction pulls in field smoothing, the structural model itself must change to (6).

### 3.2 Andersen *et al.*

AHLR simultaneously elicits utility discount rates and utility curvature. The discounting task is a choice between two rewards $x_{1}^{e}$ and $x_{2}^{e}$, spaced *τ* days apart.

Table 3 presents the MPL for τ=180 days. On each line, the earlier payment $x_{1}^{e}$ is DKK 3,000, and the later payment $x_{2}^{e}$ is something larger. Moving down the MPL, $x_{2}^{e}$ rises. With an analogy to saving in mind, this design requires the percentage increase from $x_{1}^{e}$ to $x_{2}^{e}$ to be larger than any conceivable field interest rate $r_{2}^{f}$ over the same *τ* interval.

**Table 3:** Andersen *et al*.’s MPL for a 6-month delay.

Line	$x_{1}^{e}$ (DKK)	$x_{2}^{e}$ (DKK)	APR (%)	$r_{2}^{e}$ (%)
1	3000	3075	5	2.5
2	3000	3152	10	5.1
3	3000	3229	15	7.6
4	3000	3308	20	10.3
5	3000	3387	25	12.9
6	3000	3467	30	15.6
7	3000	3548	35	18.3
8	3000	3630	40	21.0
9	3000	3713	45	23.8
10	3000	3797	50	26.6

On each line, the participant indicates a preference for the earlier or later option. That decision is governed by the comparison

$$u\left(x_{1}^{e}+\omega_{1}^{f}\right)+{\left(\frac{1}{1+\delta }\right)}^{\tau }u\left(\omega_{2}^{f}\right)⋛u\left(\omega_{1}^{f}\right)+{\left(\frac{1}{1+\delta }\right)}^{\tau }u\left(x_{2}^{e}+\omega_{2}^{f}\right)$$ (7)

where *δ* is the utility discount rate, and $\omega_{1}^{f}$ and $\omega_{2}^{f}$ are field resources. Andersen *et al*.’s version of (7) also includes a breakdown of how long the participant draws out the consumption of $x_{1}^{e}$ and $x_{2}^{e}$. Because that detail is tangential to our interference question, we focus on a special case where consumption is immediate in both periods.

As a temporal task, AHLR is easy to adapt to (1). First, we can set the discounting parameter to $\beta ={\left(\frac{1}{1+\delta }\right)}^{\tau }$. Next, we can continue the saving analogy: instead of taking $x_{1}^{e}$ during period 1, a participant can defer that amount and take $x_{2}^{e}$ in period 2. We refer to these as the “early” and “late” options.

Table 4 summarizes how those incentives translate to (1). The first option is to take the earlier payment $y_{1}^{e}=x_{1}^{e}$, thus saving $s_{1}^{e,early}=0$ and earning nothing later. The second option is to save $s_{1}^{e,late}=y_{1}^{e}$, thus taking nothing early, but earning


$$x_{2}^{e}=s_{1}^{e,late}\cdot \frac{x_{2}^{e}}{x_{1}^{e}}\;\to \;x_{2}^{e}=s_{1}^{e,late}R_{2}^{e}$$

later. The quantity $R_{2}^{e}=x_{2}^{e}/x_{1}^{e}$ is the gross increase in the payment, exactly our notion of return. In both cases, ${\tilde{y}}_{2}^{e}=0$.

**Table 4:** Translation between model (1) and AHLR.

Lifecycle Variable	AHLR “Early”	AHLR “Late”
$y_{1}^{e}$	$x_{1}^{e}$	$x_{1}^{e}$
$s_{1}^{e}$	0	$x_{1}^{e}$
${\tilde{y}}_{2}^{e}$	0	0
$R_{2}^{e}$	$x_{2}^{e}/x_{1}^{e}$	$x_{2}^{e}/x_{1}^{e}$
$y_{1}^{f}$	$\omega_{1}^{f}$	$\omega_{1}^{f}$
${\tilde{y}}_{2}^{e}$	$\omega_{2}^{f}$	$\omega_{2}^{f}$
$s_{1}^{f}$	−	−
$R_{2}^{f}$	−	−

Applying those notational changes to (7) yields the comparison

$$u\left(\left(y_{1}^{e}-s_{1}^{e,early}\right)+\omega_{1}^{f}\right)+\beta u\left(s_{1}^{e,early}R_{2}^{e}+\omega_{2}^{f}\right)⋛\;u\left(\left(y_{1}^{e}-s_{1}^{e,late}\right)+\omega_{1}^{f}\right)+\beta u\left(s_{1}^{e,late}R_{2}^{e}+\omega_{2}^{f}\right)$$ (8)

We have intentionally left alone the obvious simplifications in this expression. The reason is that (8) clearly draws out the latent smoothing variable $s_{1}^{e}$, which has a direct analog in (1). This underscores that AHLR’s core comparison does indeed involve smoothing, though it restricts the smoothing options to the extremes $s_{1}^{e}=0$ and $s_{1}^{e}=y_{1}^{e}$.

This task therefore activates the participant’s intertemporal preference *u* and discounting *β*. To provide additional utility variation outside *β*’s influence, AHLR also includes a HL task. That identification is problematic from an RU perspective, because HL’s riskiness activates *ψ*. The extra HL data generates the intended supplemental variation only when u=ψ– the EU special case.

AHLR structural estimates do indeed assume EU. If that is the correct framework, then both tasks identify the same u=ψ preference. If not, then the restriction u=ψ results in estimates that mash together risk and intertemporal attitudes.

The final quantities to reconcile are the field assets. Comparing (8) with (1), the two would match exactly if $\omega_{1}^{f}=y_{1}^{f}\;-\;s_{1}^{f}$ and ${\tilde{\omega }}_{2}^{f}={\tilde{y}}_{2}^{f}\;+\;s_{1}^{f}R_{2}^{f}$. But, because AHLR does not include field smoothing or field risk, its conception of field assets is simply $\omega_{1}^{f}=y_{1}^{f}$ and ${\tilde{\omega }}_{2}^{f}=y_{2}^{f}$. That is, only the exogenous field assets are relevant.

Indeed, Andersen *et al*. describe $\omega_{1}^{f}$ and $\omega_{2}^{f}$ as “the optimized consumption stream based on wealth and income that is perfectly anticipated before allowing for the effects of the money offered in the experimental tasks” (p. 583). In other words, the field assets are frozen in place before the experiment starts, and the experiment cannot subsequently affect them. This begs the question of endogenous resources.

The elasticity of substitution between experimental and field decisions underscores the problem with that interpretation. If the experiment truly cannot affect the field, this should be 0. But, per (4), field smoothing is likely to be highly elastic with experimental smoothing if any lifecycle asset integration is present.

### 3.3 Andreoni and Sprenger

AS elicits utility discount rates with a task more open-ended than an MPL. The participant must split a money budget *m*^*e*^ into current and future payoffs using tokens $t_{1}^{e}$ and $t_{2}^{e}$. This split takes place along the constraint


$$m^{e}=p_{1}^{e}t_{1}^{e}+p_{2}^{e}t_{2}^{e}$$

under token prices $p_{1}^{e}$ and $p_{2}^{e}$.

Unusually, AS’s structural model has Stone-Geary utility:

$$\max_{t_{1}^{e},t_{2}^{e}}\;u\left(p_{1}^{e}t_{1}^{e}-\omega_{1}^{f}\right)+\gamma \eta^{\tau }u\left(p_{2}^{e}t_{2}^{e}-\omega_{2}^{f}\right)\;\text{s.t.}\;m^{e}=p_{1}^{e}t_{1}^{e}+p_{2}^{e}t_{2}^{e}$$ (9)

The field resources $\omega_{1}^{f}$ and $\omega_{2}^{f}$ are interpreted as a minimum amount of background consumption that the participant must acquire in each period. The parameter *γ* reflects present bias, and *η* the daily utility discount factor. Those last two aspects can be reconciled with (1) by setting $\beta =\gamma \eta^{\tau }$.

Reconciling the rest requires converting token units to saving units (money). Table 5 summarizes the translation to (1). Normalizing the constraint in (9) by $p_{1}^{e}$ reveals an incentive that looks like a return:

$$\frac{m^{e}}{p_{1}^{e}}=t_{1}^{e}+\frac{p_{2}^{e}}{p_{1}^{e}}t_{2}^{e}\;\to \;{\bar{t}}_{1}^{e}=t_{1}^{e}+{\bar{R}}_{2}^{e}t_{2}^{e}$$ (10)

**Table 5:** Translation between model (1) and AS.

Lifecycle Variable	AS Variable
$y_{1}^{e}$	*m* ^*e*^
$s_{1}^{e}$	$p_{2}^{e}t_{2}^{e}$
${\tilde{y}}_{2}^{e}$	0
$R_{2}^{e}$	1
$y_{1}^{f}$	$-\omega_{1}^{f}$
${\tilde{y}}_{2}^{f}$	$-\omega_{2}^{f}$
$s_{1}^{f}$	−
$R_{2}^{f}$	−

The ratio ${\bar{R}}_{2}^{e}=p_{2}^{e}/p_{1}^{e}$ is the gross increase in the token price between the two periods. Unfortunately, this return applies to tokens, not to money as in (1).

Even though this is not the exact match we need, the normalized form (10) provides some useful clarifications that help to translate (9) into (1). First, it casts the seemingly atemporal money endowment *m*^*e*^ into a period 1 resource: the normalized endowment ${\bar{t}}_{1}^{e}$. This is the maximum possible number of period 1 tokens that could possibly be bought. It is analogous to the initial money endowment, but in tokens.

Second, the normalization shows that one of AS’s decision variables is redundant. Performing the substitution $t_{1}^{e}={\bar{t}}_{1}^{e}-{\bar{R}}_{2}^{e}t_{2}^{e}$ casts the problem purely in terms of future tokens. This is the variable that most closely resembles our saving decision. Helpfully, like the experimental saving constraint $0\leq s_{1}^{e}\leq y_{1}^{e}$, the normalized equation ensures that the number of future tokens stays in bounds: $0\leq t_{2}^{e}\leq {\bar{t}}_{1}^{e}/{\bar{R}}_{2}^{e}$.

Third, whenever it is necessary to operate in money units rather than tokens, the normalization can be undone by multiplying by $p_{1}^{e}$ throughout:


$${\bar{t}}_{1}^{e}=t_{1}^{e}+{\bar{R}}_{2}^{e}t_{2}^{e}\;\to \;p_{1}^{e}{\bar{t}}_{1}^{e}=p_{1}^{e}t_{1}^{e}+{\bar{R}}_{2}^{e}p_{1}^{e}t_{2}^{e}$$

$$0\leq t_{2}^{e}\leq \frac{{\bar{t}}_{1}^{e}}{{\bar{R}}_{2}^{e}}\;\to \;0\leq p_{1}^{e}t_{2}^{e}\leq \frac{p_{1}^{e}{\bar{t}}_{1}^{e}}{{\bar{R}}_{2}^{e}}$$

Once again, we have not made the obvious simplifications. The reason is that this denormalization suggests an analogy to monetary saving in (1): ${\bar{s}}_{1}^{e}=p_{1}^{e}t_{2}^{e}$ is the current opportunity cost of buying $t_{2}^{e}$ future tokens.

Making that substitution and simplifying, (9) becomes


$$\max_{{\bar{s}}_{1}^{e}}\;u\left(\left(m^{e}-{\bar{s}}_{1}^{e}{\bar{R}}_{2}^{e}\right)-\omega_{1}^{f}\right)+\beta u\left({\bar{s}}_{1}^{e}{\bar{R}}_{2}^{e}-\omega_{2}^{f}\right)\;\text{s.t.}\;0\leq {\bar{s}}_{1}^{e}\leq \frac{m^{e}}{{\bar{R}}_{2}^{e}}$$

Comparing this to (1), the exogenous experimental resources now match by setting $y_{1}^{e}=m^{e}$ and $y_{2}^{e}=0$. But, the endogenous resources posed in ${\bar{R}}_{2}^{e}$ terms still do not have a natural analog. The money return in (1) appears in the future alone, but this token return appears in both periods.

The monetary implications of ${\bar{R}}_{2}^{e}$ can be reconciled with (1) by normalizing $R_{2}^{e}=1$ and setting $s_{1}^{e}={\bar{s}}_{1}^{e}{\bar{R}}_{2}^{e}$:

$$\max_{s_{1}^{e}}\;u\left(\left(m^{e}-s_{1}^{e}\right)-\omega_{1}^{f}\right)+\beta u\left(s_{1}^{e}-\omega_{2}^{f}\right)\;\text{s.t.}\;0\leq s_{1}^{e}\leq m^{e}$$ (11)

This shows that AS, like AHLR, sets up a latent smoothing variable $s_{1}^{e}$. But, AS’s version does not entail a money return, since $r_{2}^{e}=0$ always. The token return ${\bar{R}}_{2}^{e}$ ultimately acts as an exchange rate between tokens and money, not as a return on saving. Importantly, in situations like AS where $r_{2}^{e}=0$, the participant’s only reason to save is to smooth out the experimental windfall $y_{1}^{e}=m^{e}$. Saving has no investment use.

(11) underscores that AS’s main source of experimental variation is *m*^*e*^. Every *m*^*e*^ results in a unique saving amount $s_{1}^{e}$, no matter the token prices. (Given $s_{1}^{e}$, the prices can be used to back out the token quantities.) Hence, changing the token prices while keeping *m*^*e*^ constant would simply pose the same question in different ways.

Because this task involves no risk, it activates only the intertemporal preference *u* and discounting *β*. It makes no statement about the other RU component, the risk preference *ψ*.

AS can be viewed as an extension of AHLR that allows participants to select their own saving amount, not just the extremes. Notably, participants seem to prefer those extremes anyway: over half of Andreoni and Sprenger’s decisions fall on boundaries. (11) suggests two reasons this might occur.

First, participants cannot use experimental saving as an investment. This eliminates one of the main reasons to save, and can lead to $s_{1}^{e}=0$. Second, if participants are on a skewed field consumption path, they will use experimental saving to force as many experimental resources as possible into the disadvantaged period. This can lead to either $s_{1}^{e}=0$ or $s_{1}^{e}=y_{1}^{e}$, depending on which period needs more support. As we show in the robustness exercises below, the balance of field and experimental incentives needed to sustain an interior $s_{1}^{e}$ is actually quite delicate.

AS’s field assets $\omega_{1}^{f}$ and $\omega_{2}^{f}$, like AHLR’s, are considered exogenous. The specification errors we discussed for AHLR therefore apply to AS as well, but the Stone-Geary form raises a new concern. Because Stone-Geary utility is not even defined before reaching the $\omega_{1}^{f}$ and $\omega_{2}^{f}$ consumption levels, those amounts are effectively exempted from smoothing. That is not the way consumption smoothing is usually understood. Indeed, when Andreoni and Sprenger estimate these quantities rather than imputing them, they do not always find negative values.

## 4 Robustness to lifecycle asset integration

Having placed HL, AHLR, and AS into the notation of (1), we next examine their robustness to various integration assumptions. This analysis involves mechanically changing levels of field resources, without paying much attention to what they mean. For some context in that regard, we begin by summarizing how the understanding of background resources in the utility function has evolved to date.

### 4.1 Background resources

In the theory of choice under static risk, decision makers evaluate their utility with respect to a terminal criterion. That conveniently compacts temporal problems into static ones whose goal resolves in a “final period.” In such settings, parameters like $\omega_{1}^{f}$ and $\omega_{2}^{f}$ are considered background wealth levels [36,38–40].

That formulation carries problematic implications. In critiques of the terminal-wealth interpretation of EU, Hansen [41] and Rabin [42] show that the assumption of reasonable risk aversion at low stakes implies absurd forms of risk aversion at high stakes. Rabin [42] and Rabin and Thaler [43] consider this inconsistency serious enough to warrant scrapping EU.

Cox and Sadiraj [44] show that this issue does not arise if utility is evaluated with respect to changes in wealth. Those changes are usually interpreted as income. As a practical matter, incorporating those changes requires adding a wealth baseline to the utility argument.

Adopting this notion of background wealth, Andersen *et al*. [9] investigate the relevance of asset integration in a static risk experiment. Their wealth baseline is defined quite broadly: it includes durable goods, real estate, and debt service; but not cash, equity in private companies, or non-tradable assets. They find that participants integrate the defined assets with experimental cash only weakly.

In the theory of lifecycle choices under risk, $\omega_{1}^{f}$ and $\omega_{2}^{f}$ are often considered background consumption levels. Experiments with a temporal aspect usually assume that asset integration originates from background consumption [16]. For example, AHLR and AS each take utility to be consumption-based rather than wealth-based, and they each include a background parameter in the utility argument.

Somewhat confusingly, consumption- and wealth-based utility are both amenable to time interpretations. For example, the multiperiod portfolio model has a terminal-wealth objective, while the multiperiod saving model has a lifecycle-consumption objective. Both have 1st order conditions that take the canonical form $E_{t}\left(m_{t+1}R_{t+1}\right)⋚1$ discussed earlier. But, their outcomes are governed by very different preference features.

It is thus critical to be able to identify a model as consumption- or wealth-based. Eeckhoudt and Schlesinger [7,45] provide a reliable way to do this, by observing what happens when an *n*^*th*^ order risk is added to the utility argument. This will activate the *n*^*th*^ derivative if utility is wealth-based, and the $n\;+\;1^{th}$ derivative if it is consumption-based. For EU (u=ψ), that reduces to whether the response to a 2nd order (variance) risk is governed by the Arrow-Pratt coefficient $-\psi^{\prime \prime }/\psi^{\prime }$, or the Kimball coefficient $-\psi^{\prime \prime }/\psi^{\prime }$.

Meyer and Meyer [46] discuss another tricky feature of the utility argument: its ability to create paradoxes in lifecycle models. For example, the equity-premium and riskfree-rate puzzles manifest as inconsistencies among intertemporal substitution, equity premia, and riskfree rates. They can be traced to artifacts of the utility specification.

The EU model at the root of the problem has isoelastic consumption utility and an isoelastic wealth value function. It also defines consumption broadly, while binding consumption tightly to wealth. That set of assumptions proves to be incompatible with US data.

When consumption is only a fraction of wealth as in the US, consumption utility can be isoelastic only if wealth utility exhibits increasing elasticity, and vice versa. Keeping both aspects isoelastic requires defining wealth more narrowly than consumption. Because that would make wealth and consumption incoherent, Meyer and Meyer recommend using utility functions that are more flexible.

RU is one route to that flexibility. The version we use has a utility-of-wealth function that ranks risky consumption possibilities via the certainty equivalent, and a utility-of-consumption function that allocates the certainty equivalent across time. These two functions admit different risk and intertemporal elasticities by default. Those domains could certainly be disentangled in other ways, but the RU method results in an intuitive SDF (3) where each attitude is governed by its own marginal rate of substitution.

### 4.2 Holt and Laury

HL implements *ψ* as an expo-power function


$$\psi \left(c\right)=\frac{1}{\alpha_{\psi }}\left[1-\exp\left(-\alpha_{\psi }\cdot \frac{c^{1-\rho_{\psi }}}{1-\rho_{\psi }}\right)\right]$$

Consistent with Meyer and Meyer’s suggestions on flexibility, the expo-power function admits increasing, decreasing, and constant RRA. Exponential utility ($\rho_{\psi }=0$) and isoelastic power utility $(\alpha_{\psi }=0$) are special cases. Our baseline calibration uses Holt and Laury’s estimates $\alpha_{\psi }=0.03$ and $\rho_{\psi }=0.73$, a combination that generates IRRA.

To be clear, this expo-power form [18,47] covers a larger parameter space than HL’s. But, HL’s analogous *a* and *r* parameters can be easily reconciled with it: $\alpha_{\psi }=a$ and $\rho_{\psi }=1-r$.

#### Exogenous field resources.

A risk-neutral participant facing the baseline MPL in Table 1 would switch from the safe lottery to the risky one at line 5. Holt and Laury’s cohort switches further down the MPL on average, signalling risk aversion. However, the position of that switchpoint is highly sensitive to the the level of exogenous resources $y_{2}^{f}$.

**Fig 1:**
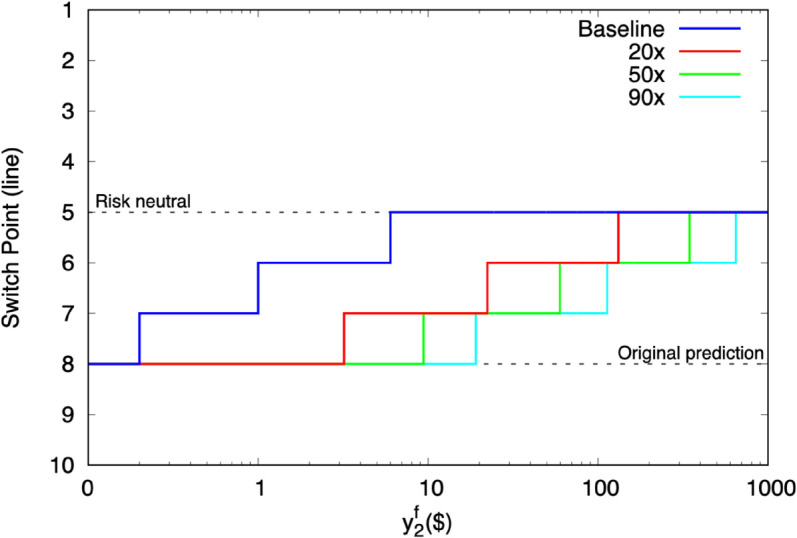
Predicted HL switchpoints as exogenous field resources increase.

Fig 1 shows how the baseline switchpoint prediction changes as $y_{2}^{f}$ increases. At HL’s assumed level $y_{2}^{f}=\text{\$}0$, the switchpoint prediction is line 8. That prediction remains intact only up to $y_{2}^{f}=\text{\$}0.10$ of exogenous resources. It falls to line 7 by $y_{2}^{f}=\text{\$}0.20$, and to line 6 by $y_{2}^{f}=\text{\$}2$. Line 5–full risk neutrality–occurs by $y_{2}^{f}=\text{\$}7$.

As a result, the HL structural estimates are probably strongly predicated on the assumption of no asset integration. But, it is hard to imagine that participants failed to consider the effects of a mere $0.20 of field resources during the task. If they did, they would have had to almost purposefully erect a mental divider between the experiment and field.

HL presents participants with a second MPL at a multiple of the original payoffs. Fig 1 repeats the baseline analysis for HL’s 20x, 50x, and 90x MPLs. This shows that interference can be postponed, but not escaped, by scaling up payoffs. Risk-neutral decisions eventually re-emerge around $y_{2}^{f}=\text{\$}100$ at 20x, and $y_{2}^{f}=\text{\$}700$ at 90x.

In exercises not shown here, we scale the exogenous field assets instead of the lottery stakes. We examine $y_{2}^{f}=\text{\$}1,000$ and $5,000, two amounts that could easily reflect a household’s monthly resources. Risk-neutral decisions occur all the way up to the 137x MPL in the first case, and up to the 611x MPL in the second.

HL’s salience thus depends strongly on the relative levels of field and experimental resources. Because HL is a risk task, this finding can be partly contextualized within the Rabin critique. Namely, as exogenous field resources push the experimental domain to higher wealth levels, participants no longer make risky experimental decisions, even though they are truly risk averse. Unlike some of Rabin’s examples, however, this interference does not require large or infinite amounts. The consequences manifest at low levels of field resources, ones that are probably consistent with real participants.

#### Endogenous field resources.

To investigate endogenous resources in HL, we must first parameterize the participant’s field smoothing environment. We consider the time interval monthly, to keep field resources relatively small. We set the baseline field return to $r_{2}^{f}=1\%$ for a similar reason.

To bound $s_{1}^{f}$’s behavior within known RU theoretical results, we limit any field risks to mean-preserving spreads (MPS). We construct an MPS starting from a balanced income stream $y_{1}^{f}=E_{1}\left({\tilde{y}}_{2}^{f}\right)$, and then create a two-outcome lottery with 50-50 probabilities centered at the future mean. Denoting the spread by $\Delta y_{2}^{f}$, the outcomes are thus $E_{1}\left({\tilde{y}}_{2}^{f}\right)\pm \Delta y_{2}^{f}$.

We must also flesh out the other two preference domains. We parameterize discounting with β=0.999, or an annual value of 0.988. Following Bostian and Heinzel [18], we build a double-expo-power specification by implementing the smoothing preference *u* with a second expo-power function:


$$u\left(c\right)=\frac{1}{\alpha_{u}}\left[1-\exp\left(-\alpha_{u}\cdot \frac{c^{1-\rho_{u}}}{1-\rho_{u}}\right)\right]$$

For *u*, the analog to RRA is the relative resistance to intertemporal substitution (RRIS), or inverse EIS. We set the baseline parameters to $\alpha_{u}=0$ and $\rho_{u}=2$, an isoelastic utility function in the neighborhood of macroeconomic estimates.

We illustrate interference from endogenous field resources with the 20x MPL. We set field income levels to $y_{1}^{f}=E_{1}\left({\tilde{y}}_{2}^{f}\right)=\text{\$}100$, and omit field risk for clarity (${\Delta y}_{2}^{f}=0$). We assume first that no field smoothing instrument exists, and then that a field smoothing instrument exists and pays returns of $r_{2}^{f}=1\%$ and 10%.

Table 6 presents the switchpoints under each assumption, as well as the field saving amounts $s_{1}^{f,safe}$ and $s_{1}^{f,risky}$ latent on each line. Several features of this table are important. First and foremost, just by assuming that the participant has some form of field smoothing instrument, the switchpoint prediction rises from line 8 to line 6.

**Table 6:** HL 20x decisions with and without a field saving instrument ($y_{1}^{f}=y_{2}^{f}=\text{\$}100$).

	No field saving		$r_{2}^{f}=1\%$		$r_{2}^{f}=10\%$	
Line	$s_{1}^{f,safe}$	$s_{1}^{f,risky}$	$s_{1}^{f,safe}$	$s_{1}^{f,risky}$	$s_{1}^{f,safe}$	$s_{1}^{f,risky}$
1	0	0	33.60	46.66	33.50	45.71
2	0	0	33.22	44.32	33.15	43.46
3	0	0	32.84	41.81	32.79	41.07
4	0	0	32.45	39.10	32.43	38.49
5	0	0	32.06	36.16	32.06	35.69
6	0	0	31.66	32.91	31.68	32.61
7	0	0	31.26	29.25	31.30	29.15
8	0	0	30.84	24.99	30.92	25.13
9	0	0	30.43	19.68	30.53	20.18
10	0	0	30.00	11.64	30.13	12.91
Switchpoint	8		6		6	

To reiterate, when field smoothing is impossible, the participant tolerates period 2’s lopsided but risk-averse consumption outcome. But, when field smoothing is allowed, the participant seemingly becomes *less* risk averse. This is very strange: we have not touched *ψ* (or any other preference domain), and so we know for a fact that the participant’s risk attitude is the same in both settings.

The prediction changes because the field smoothing instrument allows the participant to re-optimize the consumption-smoothing and precautionary motives as risk is manipulated. This “hedge across time” involving all three preferences is more powerful than the simple static hedge afforded by risk aversion. It lets the participant engage in more MPL risk.

As our theoretical discussion suggested, each side of (6) does indeed yield different amounts of field saving. Because HL alters the mean and variance of the consumption path simultaneously, the increase from $s_{1}^{f,safe}$ to $s_{1}^{f,risky}$ on each line is a combined product of the consumption-smoothing and precautionary motives. The change in consumption smoothing is triggered by the mean difference between ${\tilde{y}}_{2}^{e,safe}$ and ${\tilde{y}}_{2}^{e,risky}$, while the change in precaution is triggered by the variance difference. The latter entails supporting a risky period by saving more as risk rises, which can be seen moving up the MPL.

Thus, HL decisions are also sensitive to whether the participant has a field smoothing instrument. A participant who seems to be risk neutral per the MPL may be truly risk averse, but re-hedging their lifecycle outside the experimenter’s view.

#### Other considerations.

**Fig 2:**
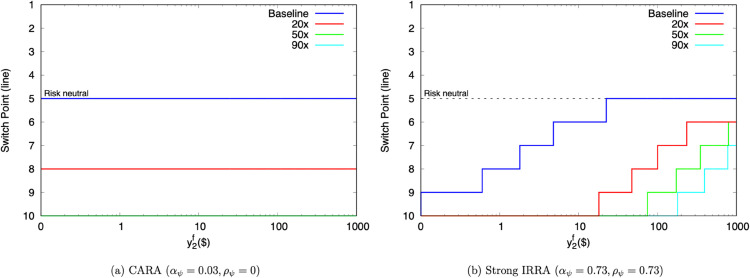
Predicted HL switchpoints for other ψ specifications.

Fig 2 re-examines interference from exogenous field resources under two extreme risk attitudes. These exercises illustrate how $\alpha_{\psi }$ and $\rho_{\psi }$ act together to explain decisions at very different payoff scales.

The left panel plots decisions under CARA. For this attitude, the only facet that matters is ${\tilde{y}}_{2}^{e}$’s payoff level. Because that is fixed for a given scaling, and because $y_{2}^{f}$ carries no risk, the switchpoint within a scaling never varies with $y_{2}^{f}$. The switchpoint at the baseline scaling is line 5, indistinguishable from risk neutrality. The 20x scaling now yields the original switchpoint at line 8. No switches ever occur at 50x and 90x. CARA thus implies a huge amount of risk aversion at large stakes.

The right panel repeats this exercise for much stronger IRRA. Unlike the CARA switchpoints, these do eventually change as $y_{2}^{f}$ rises. So, $\rho_{\psi }$ serves to tamp down $\alpha_{\psi }$’s explosiveness at large amounts, while still generating risk aversion at small amounts.

### 4.3 Andersen *et al.*

Andersen *et al*.’s parameter estimates are an isoelastic *u* with $\rho_{u}=0.74$, and an annual pure rate of time preference of 10%. We examine interference using the APR structure in Table 3, at durations of 1, 3, 6, and 12 months. We build a two-period environment by breaking each interval into two equally long segments.

To keep units uniform across examples, we convert DKK to USD at 6.55 to 1, Andersen *et al*.’s reported exchange rate. Thus, $y_{1}^{e}$ is about $450. Because *u* is isoelastic, multiplicatively scaling its argument by an exchange rate will not alter any switchpoint predictions.

#### Exogenous field resources.

**Fig 3:**
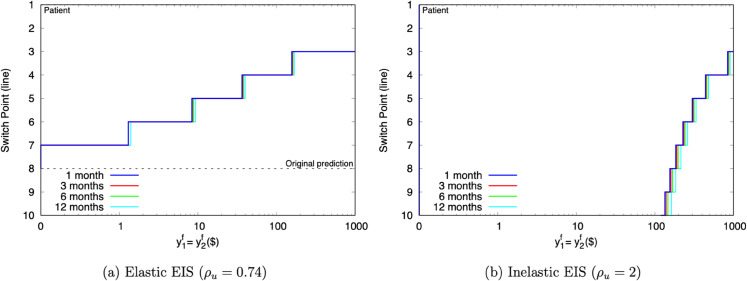
Predicted AHLR switchpoints as exogenous field resources increase.

Fig 3 presents the switchpoint predictions as exogenous field resources increase. Andersen *et al*. calibrate their exogenous field resources to a government survey that finds average daily field consumption at DKK 118. In Fig 3’s units, a day’s worth of background consumption is about $20, and three months’ worth about $1,600.

The switchpoints in the left panel are certainly sensitive to $y_{1}^{f}$ and $y_{2}^{f}$. The switchpoint moves from line 8 at $y_{1}^{f}=y_{2}^{f}=\text{\$}0$ to line 3 at $y_{1}^{f}=y_{2}^{f}=\text{\$}1,000$. That prediction evolves in essentially the same way at different *τ* intervals.

Because Andersen *et al*. estimate *u* in conjunction with a HL task, it is not entirely surprising they find a $\rho_{u}$ close to Holt and Laury’s $\rho_{\psi }$ (0.74 vs. 0.73). But, it is important to remember that those parameters address different attitudes: $\rho_{u}$ measures *u*’s RRIS, while $\rho_{\psi }$ measures *ψ*’s RRA. Thus, the same numerical estimate signals something different in AHLR’s intertemporal context than it does in HL’s static context. For AHLR, this estimate means that RRIS is 0.74, and so EIS is 1/0.74 = 1.35.

This juxtaposition underscores that it is not possible to extinguish the distinction between RRA and RRIS by making them “mathematically equivalent” via the u=ψ restriction. In intertemporal settings, that equivalence simply collapses RU to EU. It does not somehow compress RRA and RRIS into a single attitude. Both are still present under EU–they are just forced to take the same value.

Elastic EIS is hardly ever found in macroeconomic data. So, the right panel of Fig 3 repeats this exercise under $\rho_{u}=2$, the same inelastic value used for our HL analysis. These switchpoint predictions move much more sharply than the elastic ones. They do not even dislodge from the bottom of the MPL until about $y_{1}^{f}=y_{2}^{f}=\text{\$}100$, but they still reach line 3 by $y_{1}^{f}=y_{2}^{f}=\text{\$}1,000$. There is slightly more separation in the predictions at different time intervals, but they largely move in tandem as before.

The key takeaway is that many switchpoint predictions can be rationalized under either elasticity assumption. For example, line 5 arises at about $y_{1}^{f}=y_{2}^{f}=\text{\$}10$ under the elastic *u*, and at about $y_{1}^{f}=y_{2}^{f}=\text{\$}300$ under the inelastic *u*. This illustrates that AHLR, like HL, is sensitive to the level of exogenous resources. In this case, as field resources increase, the participant appears to be more patient.

Unlike HL, this loss of salience cannot be attributed to the Rabin critique. Because AHLR has no risk, it evades that concern. Instead, it is wholly a consequence of the participant using the experiment to smooth out field consumption. When field resources are large enough, the desired smoothness results in always choosing the early option.

#### Endogenous field resources.

Table 7 presents the switchpoint predictions for the 6-month MPL, with and without the assumption that the participant has a field smoothing instrument. As with the HL analysis, we set field returns to $r_{2}^{f}=1\%$ and 10%. We set field resources to $y_{1}^{f}=E_{1}\left(y_{2}^{f}\right)=\text{\$}500$, and continue to omit field risk ($\Delta y_{2}^{f}=0$).

**Table 7:** AHLR 6-month decisions with and without a field saving instrument ($y_{1}^{f}=y_{2}^{f}=\text{\$}500$).

	No field saving		$r_{2}^{f}=1\%$		$r_{2}^{f}=10\%$	
Line	$s_{1}^{f,early}$	$s_{1}^{f,late}$	$s_{1}^{f,early}$	$s_{1}^{f,late}$	$s_{1}^{f,early}$	$s_{1}^{f,late}$
1	0	0	209.37	-252.19	240.82	-201.37
2	0	0	209.37	-258.14	240.82	-206.76
3	0	0	209.37	-264.16	240.82	-212.21
4	0	0	209.37	-270.26	240.82	-217.72
5	0	0	209.37	-276.43	240.82	-223.31
6	0	0	209.37	-282.67	240.82	-228.96
7	0	0	209.37	-288.99	240.82	-234.67
8	0	0	209.37	-295.38	240.82	-240.45
9	0	0	209.37	-301.84	240.82	-246.30
10	0	0	209.37	-308.37	240.82	-252.21
Switchpoint	3		1		4	

As in HL, the AHLR switchpoint predictions change just by assuming that the participant has a field smoothing instrument. The prediction in the absence of field smoothing is line 3. This rises to line 1 when $r_{2}^{f}=1\%$, and actually falls to line 4 when $r_{2}^{f}=10\%$.

Also like HL, field smoothing changes markedly on each side of the AHLR comparison (8). The value of $s_{1}^{f}$ is always positive for the early option, indicating that the participant will smooth forward some of the early experimental windfall. Similarly, $s_{1}^{f}$ is always negative for the late option, indicating that the participant will smooth back some of the late windfall.

The move from line 3 to line 1 when $r_{2}^{f}=1\%$ makes the participant appear more patient. But, because we have not changed anything about preferences, we know that this outcome is instead rooted in some sort of substitution. In this case, the ability to smooth in the field allows the participant to support a seemingly more patient outcome in the MPL. That “patience” is nothing more than opting for the larger late experimental payment, and smoothing some of it back with the field instrument.

Those same attitudes are present when $r_{2}^{f}=10\%$. But, this situation taps another consideration: the high return on field saving makes it a very attractive investment. That factor causes the participant to opt for the earlier experimental payment, and save it with the field instrument. The switchpoint thus moves down the MPL instead of up.

So, the mere ability to smooth in the field can also result in different AHLR predictions. Saving’s smoothing use can be triggered with fairly small field returns. Larger returns can also pull in saving’s investment use.

#### Other considerations.

Even though AHLR does not contain risk itself, a participant could certainly bear field risk. We explore those implications by again setting $y_{1}^{f}=E_{1}\left(y_{2}^{f}\right)=\text{\$}500$, and varying the MPS spread $\Delta y_{2}^{f}$ from $0 to $500. We assume away endogenous field resources for clarity.

Because this set of incentives will activate both risk and smoothing preferences, we use a full RU specification. We set *ψ* to Holt and Laury’s IRRA estimates, and *u* to the previous power function with $\rho_{u}=2$. To reiterate our earlier warning about the difference between RRA and RRIS, it would be unwise to impute *u* with Holt and Laury’s estimates. Those values would imply IRRIS, and eventually send EIS all the way to 0.

**Fig 4:**
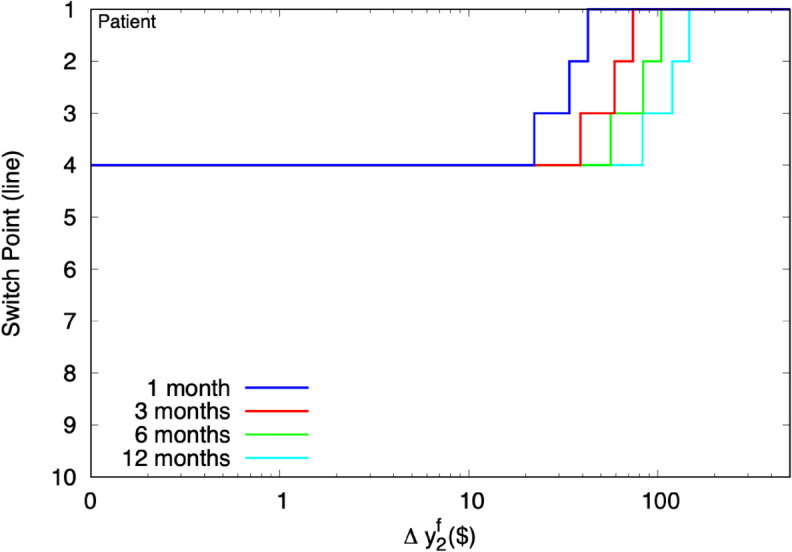
Predicted AHLR switchpoints under RU, for MPS of ${\tilde{y}}_{2}^{f}(y_{1}^{f}=E_{1}\left({\tilde{y}}_{2}^{f}\right)=\text{\$}500)$.

Fig 4 plots the switchpoints. As $\Delta y_{2}^{f}$ increases, the switchpoint moves up the MPL. Because this exercise involves only changes in risk, that outcome is fully attributable to the participant’s precautionary motive, which supports higher future risk by saving more. In the AHLR context, “saving more” means “choosing the late option.”

Because participants can potentially use experimental smoothing to mitigate field risk, the experimenter should also have a grasp on how much field risk participants face. Higher field risk will lead to more experimental smoothing.

### 4.4 Andreoni and Sprenger

For easy comparison with the AHLR results, we examine AS interference by imputing incentives from Table 3: $m^{e}=\text{DKK }3,000$ at a 6-month interval.

#### Exogenous field resources.

**Fig 5:**
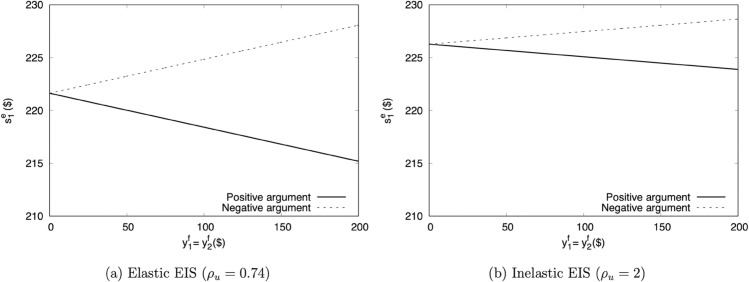
Predicted AS saving amounts when exogenous field resources are construed as positive or negative.

An obvious difference between AHLR and AS is the latter’s Stone-Geary treatment of exogenous field resources. Fig 5 plots the predicted AS saving amounts $s_{1}^{e}$ assuming that $y_{1}^{f}$ and $y_{2}^{f}$ can be positive or negative. This shows that Stone-Geary inverts the standard relationship between saving and field resources.

The downward-sloping lines reflect the usual understanding, where the propensity to save falls as the participant holds additional positive resources. This occurs because those extra resources push the consumption interval away from the highly curved parts of *u*, thereby diminishing the desire to smooth.

The upward-sloping lines reflect the Stone-Geary conception. Because the movements along the utility function now operate in reverse, the propensity to save rises. As the participant receives additional negative resources, a greater part of the consumption interval falls into the very curved areas of *u*. Those areas reflect undesirably lumpy outcomes, and so the participant saves more to smooth out the consumption path.

AS sets $r_{2}^{e}=0$, eliminating experimental saving’s investment use. Fig 5 shows that participants will save anyway. This underscores that saving does have a meaningful smoothing function separate from its investment function. Unfortunately, Fig 5 also shows that the field resources $y_{1}^{f}$ and $y_{2}^{f}$ can once again interfere with the experimental decision $s_{1}^{e}$. As the participant’s field resources increase, the desire to save in the experiment falls.

#### Endogenous field resources.

**Fig 6:**
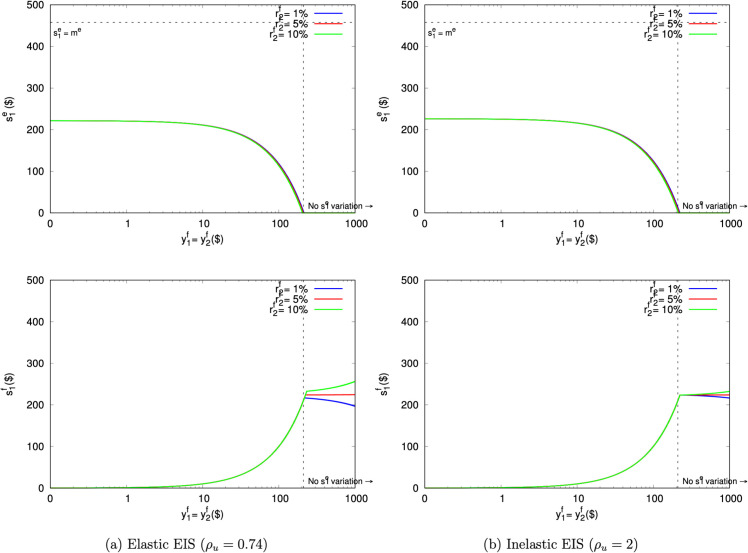
Predicted AS saving amounts when a field saving instrument is present.

Fig 6 repeats this exercise assuming that the participant has field smoothing instruments that return $r_{2}^{f}=1\%$, 5%, and 10%. These plots provide the clearest illustration yet of the elasticity (4) between experimental and field smoothing: whenever $s_{1}^{e}$ falls, $s_{1}^{f}$ simultaneously rises to compensate.

Because $r_{2}^{f}$ is always greater than $r_{2}^{e}$ in Fig 6, that tradeoff always moves in the direction of substituting experimental saving with field saving. Importantly, the fact that field saving has a higher return does not necessarily mean that the participant will forego experimental saving. Instead, up to about $y_{1}^{f}=y_{2}^{f}=\text{\$}200$, the participant saves with both instruments. That mixture lets the participant trade some experimental saving for better-returning field saving, while also keeping the consumption path sufficiently smooth.

Past $y_{1}^{f}=y_{2}^{f}=\text{\$}200$, the participant does forego experimental saving. In fact, the participant would like to borrow from the experimenter and save those funds in the field, but the design forbids it. Thus, a participant with more than $200 of field resources will always choose $s_{1}^{e}=0$ in AS. Critically, the experimenter will not be able to determine ex post whether that boundary outcome has been generated by the participant’s preferences (e.g., strong impatience or high RRIS) or by this interference. Those explanations are observationally equivalent.

**Fig 7:**
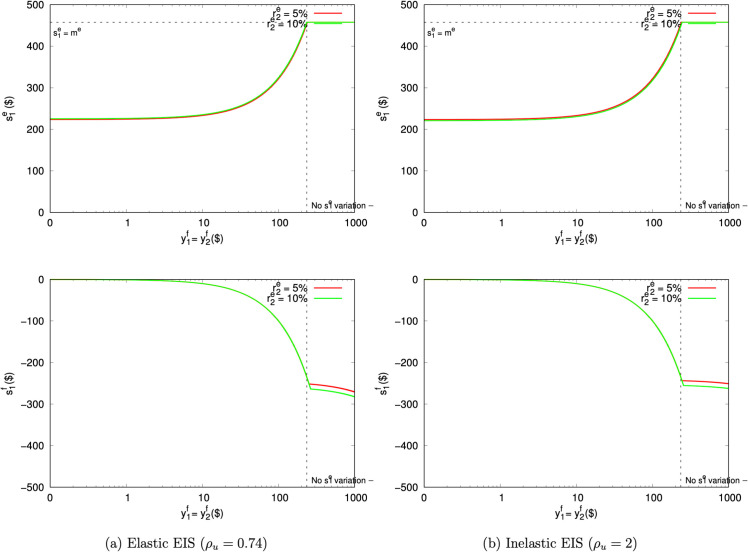
Predicted AS saving amounts if $s_{1}^{e}$ has a return ($r_{2}^{f}=1\%$).

Elasticity (4) can cause problems at the upper bound just as easily. Fig 7 presents a hypothetical exercise that modifies the AS task to pay experimental returns $r_{2}^{e}=5\%$ and 10%, while the participant’s field return is $r_{2}^{f}=1\%$. The experiment is now a much better investment than the field, and so the MRS operates in reverse. Experimental saving reaches its upper bound $s_{1}^{e}=m^{e}$ at about $y_{1}^{f}=y_{2}^{f}=\text{\$}200$. The participant compensates for higher experimental saving by borrowing in the field.

AS thus is sensitive to endogenous field resources in a way that naturally results in boundary decisions. That interference can result in higher or lower experimental saving. The former is like the attenuation towards patient outcomes that occurs with AHLR.

## 5 Discussion

This paper overlays a thought experiment onto the design of risk and time experiments: what would happen if participants treated an experiment not as a wholly isolated task, but as an injection of resources into their existing lifecycle plans? In the three classic designs we examine–encompassing static and dynamic tasks; risk and time manipulations; and menu and free responses– experimental decisions change markedly when participants are assumed to trade off experiment and field resources, versus not. In other words, inferences in these experiments are not very robust to lifecycle asset integration. Moreover, because field interference occurs reliably, and with small amounts, we strongly suspect this problem extends beyond these three settings.

We reach those conclusions by casting each experiment’s incentives into a meta-model that represents a participant’s unified experiment-field environment. The two-period framework underpinning this model is a workhorse in intertemporal choice theory, with well-studied properties. Those properties make theoretically standard, but situationally remarkable, predictions on how easily lifecycle asset integration can interfere with experiments.

The attending inference issues can be illustrated with a simple AHLR example. Suppose an experimenter observes switchpoints at rows 5, 6, and 7 of the AHLR menu. Under the standard interpretation, the row 5 switchpoint reflects the most patient preferences, the row 7 switchpoint reflects the least patient preferences, and the row 6 switchpoint reflects preferences in between. In other words, switching further down the menu implies less patience.

But, once lifecycle asset integration is admitted as a possibility, the experimenter faces a quandary. Per our analysis, those same switchpoints can now be interpreted in three conflicting ways:

1. If participants have not integrated anything, the standard interpretation is correct.
2. If participants have integrated exogenous field resources, rows 5-7 will reflect preferences that are less patient than the standard interpretation. Very problematically, the straightforward relationship between rows and patience will almost surely break if different participants have integrated different amounts. For example, a participant with low patience and moderate integration could switch at row 5, while one with similarly low patience but little integration still switches at row 7. Intertemporal preferences, which smooth field and experimental resources in tandem, are responsible for this divergence.
3. If participants have integrated endogenous field resources, rows 5-7 will reflect preferences that are either more or less patient than the standard interpretation, depending on the size of the field return. When that return is small, issues like the ones for exogenous resources will arise. When that return is large, rows further down the table might actually imply more patience. For example, a participant with high patience and moderate integration could switch at row 7, while one with similarly high patience but little integration still switches at row 5. Intertemporal preferences are responsible once again, but the intertemporal tradeoffs are complicated by the fact that the field return makes a good investment.

These three interpretations are observationally equivalent: an experimenter will not be able to tell to what extent the switchpoint data have been generated by the preferences targeted by the task, or by lifecycle asset integration. We find similar observational equivalence in the static HL risk task, and the free-response AS saving task. Field resources on the scale of $100 to $1,000 almost always cause these problems, but resources on the scale of $10 can too. The usual outcome is a kind of attenuation bias pulling data toward risk neutral and patient interpretations, even though preferences are solidly risk averse and impatient.

The lone bit of prima facie evidence that we find for lifecycle asset integration is large numbers of boundary decisions. Because asset integration involves substituting between experimental and field resources, it is natural for experimental incentives that are quite stingy or quite rich relative to the field to result in minimal or maximal uptake (see Fig A in S1 Appendix). It may hence be especially tricky to elicit interior decisions in free-response designs like AS. We ourselves have dismissed substantial boundary decisions in experimental runs as a chance design flaw. In light of these results, that flaw might be very specific.

In sum, small field amounts can cause big experimental inference problems, and there is not much that can be gleaned about the extent of those problems from just a cursory glance at the data. In light of that, we are reluctant to offer suggestions on “testing for” lifecycle asset integration. In our view, “testing” understates the severity of the issue. Per our results, if lifecycle asset integration is suspected, design and control strategies *absolutely must* be implemented ex ante and ex post in order for an experiment to be successful.

Ex ante, our model’s key lesson is that experimenters should undertake design strategies to fend off asset integration’s inexorable pull toward neutrality. One mitigation that makes universal sense is pre-screening participants, to give experimenters a better chance to match incentives to participants’ field environments. Poorly-matched incentives are predestined to yield little data variation (see Figs 1, 4, and 6), and such datasets are ineffectual for any kind of ex post analysis. The pre-screen will require attention to empirical details on participants’ exogenous and endogenous assets, and how those interfere with the design under consideration.

If a design does generate useful variation, experimenters should then bear in mind ex post that attenuation from lifecycle asset integration has reduced the sensitivity of their statistical tests. This will shrink the differentials underpinning basic comparison tests, and promote false negatives. Reduced-form controls that mirror the empirical details in the pre-screen will be needed to address that bias.

If preference estimates are also needed, the structural equations should start from something akin to joint system (2a) and (2b), which captures the entire experiment-field interaction. (When an experiment does not include a smoothing decision, a single equation like (2a) is enough.) This is a much taller order than the oft-used method of eliciting preferences on a “one task per attitude” basis, and then rolling the data into a structural likelihood like


likelihood=smoothing likelihood+risk-aversion likelihood+discounting likelihood

Besides omitting the field, this method weighs all three preferences equally, but those preferences do not actually have equal weights in the decision model (1) generating the data. SDF (3), embedded in (2a) and (2b), inherently provides the correct weighting.

Bostian and Heinzel [18] underscore the helpfulness of that weighting in numerical decompositions of $s_{1}^{*}$ into its *u*-mediated smoothing motive and *ψ*-mediated precautionary motive. These suggest that more than 80% of $s_{1}^{*}$ is attributable to *u*–even when risks are very large. The reason can be seen by perturbing the SDF:


$$\frac{\partial \text{SDF}}{\partial s_{1}}=-\text{SDF}[\left(ARIS_{u}\left(CE\left({\tilde{c}}_{2}\right)\right)CE^{\prime }\left({\tilde{c}}_{2}\right)+ARIS_{u}\left(c_{1}\right)\right)\;-\left(ARA_{\psi }\left(CE\left({\tilde{c}}_{2}\right)\right)CE^{\prime }\left({\tilde{c}}_{2}\right)-ARA_{\psi }\left({\tilde{c}}_{2}\right)R_{2}\right)]$$

To illustrate with isoelastic preferences on a balanced consumption path, $\partial \text{SDF}/\partial s_{1}\approx -\text{SDF}\left(\rho_{u}-\rho_{\psi }\right)$. So, no matter how the field and experiment are configured, much of the behavioral content of $s_{1}^{f}$ and $s_{1}^{e}$ is governed by the simple difference between *ARIS*_*u*_ and $ARA_{\psi }$. Because most evidence puts $ARIS_{u}>ARA_{\psi }$, smoothing preferences will likely dominate $s_{1}^{f}$ and $s_{1}^{e}$ by default. For an experimenter investigating risk preferences, the key takeaway is that the observational unit ($s_{1}^{e*})$ can be contaminated by a behavioral confound (*ARIS*_*u*_) that is potentially several times stronger than the behavior of interest $\left(ARA_{\psi }\right)$.

As a word of caution, many structural models assume EU. In our experience, this opens up the experimenter to confusing RRA and RRIS, particularly when an assortment of tasks on risk and time has been assembled together. Helpfully, the RU formulation of (1) makes plain that some descriptions of risk aversion just do not make sense as elasticities of intertemporal substitution. For example, Holt and Laury’s IRRA estimate would not make a good RRIS, because IRRIS eventually implies zero intertemporal elasticity.

The benefits of an RU stance toward modeling are not rooted in the RU functional form per se, but in contextualizing experimental design within the trio of discounting, risk aversion, and intertemporal substitution by default. Lifecycle asset integration will activate all of those preferences at once–no matter what preference the task is ostensibly targeting–and RU-style framing faithfully captures that. It also clarifies which field data would serve as appropriate controls for the resulting interference. If, after viewing an experiment in this light, the experimenter determines that simplifications are warranted (e.g., EU or a smaller set of field resources), those can certainly be made consistently within the framework.

Each of our examples is a “laboratory experiment” in the sense of its having been originally conducted in a laboratory. Because laboratory experiments cleanly segregate the experimenter’s manipulation from all other incentives, they fit our meta-model neatly. But, our model’s explicit link between experiment and field also calls out an uncomfortable limitation of the laboratory paradigm: a laboratory room does not necessarily wall off the field. Unquestionably, participants can mentally incorporate their own field resources into a task conducted inside a laboratory.

This, in turn, suggests that the standard taxonomy’s distinctions between laboratory, field, artefactual, and natural experiments [48] start to blur for risk and time designs. Indeed, our analysis shows that trying to make those designs “more realistic” with large, field-scale incentives will not change anything. Field-scale lifecycle asset integration will intrude in those situations too. Because interference can occur at any scale, experimenters should have a good understanding of their participants’ field resources when implementing these tasks.

This becomes even more essential if the relevant incentives are non-financial. For instance, a farmer participating in an experiment in a developing country might use grain storage as a field smoothing instrument, instead of money. Our framework can still work in this situation, but grain-equivalent incentives must be meticulously incorporated.

These experiments’ prior successes may be linked to their subject pools, usually students or similar groups with low amounts of lifecycle assets. Our model contains this scenario as a special case, and confirms that designs will largely work as planned when field resources are low. But, it also underscores that the external validity of this case should not be reflexively inflated, because asset integration can intrude easily. Neglecting the diversity of lifecycle assets across cohorts could be a source of replication failure [49].

Our unified experiment-field framework can help prevent that outcome. We have illustrated how to cast three classic experiments into our model with simple changes of notation; others can probably be accommodated similarly. Once a design is so embedded, experimenters can repeat the same exercises we perform, to assess how lifecycle asset integration might interfere in their own contexts.

## Supporting information

S1 AppendixDerivation of 1st order conditions and elasticity of substitution between experimental and field decisions $\epsilon^{e,f}$.(PDF)
