# Supplementary material for: Robustness of inferences in risk and time experiments to lifecycle asset integration
Source: PLoS One. 2025 Sep 29;20(9):e0332888. doi: 10.1371/journal.pone.0332888 (PMC12478945; doi:10.1371/journal.pone.0332888)
Supplement: S1 Appendix — (PDF) [file pone.0332888.s001.pdf]

S1 Appendix:

Derivation of 1st order conditions and elasticity of substitution between experimental and field decisions $\epsilon^{e,f}$

A 1st order conditions

The full Lagrangian underpinning Eq (1) is

$$\begin{aligned}
L \equiv\ & u\left(c_1^f\right) + \beta u\left(CE\left(\tilde{c}_2^f\right)\right) \\
& + \mu_1\left(y_1^f + y_1^e - c_1^f - s_1^f - s_1^e\right) \\
& + \mu_2\left(\tilde{y}_2^f + \tilde{y}_2^e + s_1^f R_2^f + s_1^e R_2^e - \tilde{c}_2^f\right) \\
& + \lambda_1 c_1^f \\
& + \lambda_2 \tilde{c}_2^f \\
& + \lambda^f\left(s_1^f - \tilde{y}_2^f\right) \\
& + \upsilon^f\left(y_1^f - s_1^f\right) \\
& + \lambda^e s_1^e \\
& + \upsilon^e\left(y_1^e - s_1^e\right)
\end{aligned}$$

The choice variables are c_1^f, $\tilde{c}_2^f$, s_1^f, and s_1^e. Instead of relying on Kuhn-Tucker shortcuts, L spells out every Lagrange multiplier. We will shortly collapse the choice variables to just s_1^f and s_1^e, but starting from this perspective illustrates why that simplification is warranted.

L's 1st order conditions are

$$\begin{aligned}
c_1^f &: u'\left(c_1^f\right) - \mu_1 + \lambda_1 && = 0 \\
c_2^f &: \beta u'\left(CE\left(\tilde{c}_2^f\right)\right) CE'\left(\tilde{c}_2^f\right) - \mu_2 + \lambda_2 && = 0 \\
s_1^f &: -\mu_1 + \mu_2 R_2^f + \lambda^f - \upsilon^f && = 0 \\
s_1^e &: -\mu_1 + \mu_2 R_2^e + \lambda^e - \upsilon^e && = 0 \\
\mu_1 &: y_1^f + y_1^e - c_1^f - s_1^f - s_1^e && \geq 0 \\
\mu_2 &: \tilde{y}_2^f + \tilde{y}_2^e + s_1^e R_2^e + s_1^f R_2^f - \tilde{c}_2^f && \geq 0 \\
\lambda_1 &: c_1^f && \geq 0 \\
\lambda_2 &: \tilde{c}_2^f && \geq 0
\end{aligned}$$

$$\begin{aligned}
\lambda^f &: s_1^f - \tilde{y}_2^f && \geq 0 \\
\upsilon^f &: y_1^f - s_1^f && \geq 0 \\
\lambda^e &: s_1^e && \geq 0 \\
\upsilon^e &: y_1^e - s_1^e && \geq 0
\end{aligned}$$

The properties of u and ψ that satisfy L's 2nd order conditions (Gollier [28]) also guarantee $c_1^{f*}, \tilde{c}_2^{f*} > 0$, and that the two resource constraints hold with equality. Thus, $\mu_1^{f*}, \mu_2^{f*} > 0$ and $\lambda_1^*, \lambda_2^* = 0$. Because the consumption path is then fully determined by state variables and saving amounts, c_1^f and $\tilde{c}_2^f$ are revealed as redundant choice variables.

Applying those simplifications yields the shorter Lagrangian

$$\begin{aligned}
\mathcal{L} \equiv\ & u\left(y_1^f + y_1^e - s_1^f - s_1^e\right) + \beta u\left(CE\left(\tilde{y}_2^f + \tilde{y}_2^e + s_1^f R_2^f + s_1^e R_2^e\right)\right) \\
& + \lambda^f\left(s_1^f - y_2^f\right) \\
& + \upsilon^f\left(y_1^f - s_1^f\right) \\
& + \lambda^e s_1^e \\
& + \upsilon^e\left(y_1^e - s_1^e\right)
\end{aligned}$$

with 1st order conditions

$$\begin{aligned}
s_1^f &: -u'\left(c_1^f\right) + \beta u'\left(\tilde{c}_2^f\right) CE'\left(\tilde{c}_2^f\right) R_2^f + \lambda^f - \upsilon^f && = 0 \\
s_1^e &: -u'\left(c_1^f\right) + \beta u'\left(\tilde{c}_2^f\right) CE'\left(\tilde{c}_2^f\right) R_2^e + \lambda^e - \upsilon^e && = 0 \\
\lambda^f &: s_1^f - \tilde{y}_2^f && \geq 0 \\
\upsilon^f &: y_1^f - s_1^f && \geq 0 \\
\lambda^e &: s_1^e && \geq 0 \\
\upsilon^e &: y_1^e - s_1^e && \geq 0
\end{aligned}$$

Conveniently, $\mathcal{L}$ recasts L's consumption-saving tradeoffs in terms of saving decisions alone. This is the mathematical motivation for Eq (1).

The Lagrange multipliers on the saving variables cannot be characterized in general. However, because experimental incentives are typically smaller than field incentives, we would usually expect s_1^{f*} to be interior to lifecycle field income, implying $\lambda^{f*}, \upsilon^{f*} = 0$. But, that assumption could certainly fail in some circumstances (e.g., an experiment conducted in a developing country where y_1^e is several months' field income).

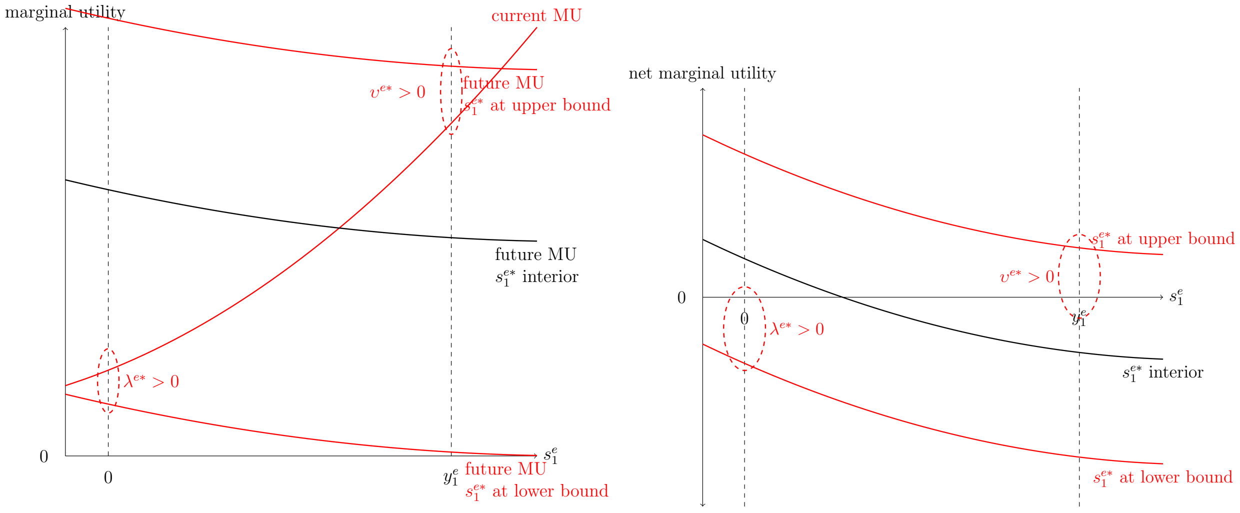

(a) Current and future MU

(b) Net MU

Fig A: Two visualizations of λ^e and υ^e using the marginal utilities from s_1^e's 1st order condition

To illustrate how this setup admits boundary values of s_1^e, Fig A presents two related visualizations of Lagrange multipliers λ^e and υ^e. The first is rooted in an intuitive decomposition of s_1^e's 1st order condition into its current and future marginal utility (MU) components, plotted as a supply-and-demand system (Carroll and Kimball, 2005; Bostian and Heinzel [18]). Under this reading, the participant's future self demands resources via saving, which its current self supplies.

The second visualization is the 1st order condition itself. Given the supply-and-demand reading, this can be viewed as the net surplus from the participant's mental trade.

Per Fig A, $s_1^{e*} \in [0, y_1^e]$ will be interior only when the participant's current self can exactly supply the saving that its future self demands (i.e, when net surplus is 0). If current MU is instead higher than future MU everywhere in the allowed interval, the participant will choose $s_1^{e*} = 0$, because the utility gap $\lambda^{e*} > 0$ is largest on the left boundary. Conversely, if future MU is always higher than current MU everywhere in the allowed interval, the participant will choose $s_1^{e*} = y_1^e$, because the utility gap $\upsilon^{e*} > 0$ is largest on the right boundary.

These illustrative utility gaps have been generated by shifting future MU alone. Such shifts could reflect simple differences in y_2^e, y_2^f, R_2^e, or R_2^f. But, to be clear, those are not the only ways to produce boundary decisions in this model. In general, any set of experimental incentives that is too stingy relative to the field will yield the left boundary $s_1^{e*} = 0$, and any that is too rich will yield the right boundary $s_1^{e*} = y_1^e$.

B Elasticity of substitution $\epsilon^{e,f}$

The elasticity Eq (4) of s_1^f with respect to s_1^e can be developed from the usual definition

$$\epsilon^{e,f} \equiv \frac{d\ln\left(s_1^f\right)}{d\ln\left(s_1^e\right)} = \frac{ds_1^f}{ds_1^e} \cdot \frac{s_1^e}{s_1^f}$$

The derivative ds_1^f/ds_1^e can be extracted from $\mathcal{L}$'s 1st order conditions. Summing the s_1^f and s_1^e conditions yields

$$\mathcal{O} \equiv -u'\left(c_1^f\right) + \beta u'\left(\tilde{c}_2^f\right) CE'\left(\tilde{c}_2^f\right)\frac{1}{2}\left(R_2^f + R_2^e\right) + \frac{1}{2}\left(\lambda^f - \upsilon^f\right) + \frac{1}{2}\left(\lambda^e - \upsilon^e\right) = 0$$

The derivative follows by applying the implicit function theorem to $\mathcal{O}$:

$$\frac{ds_1^f}{ds_1^e} = -\frac{\partial\mathcal{O}/\partial s_1^e}{\partial\mathcal{O}/\partial s_1^f}$$

where

$$\frac{\partial\mathcal{O}}{\partial s_1^f} = u''\left(c_1^f\right) + \beta\left[u''\left(\tilde{c}_2^f\right) CE'\left(\tilde{c}_2^f\right)^2 + u'\left(\tilde{c}_2^f\right) CE''\left(\tilde{c}_2^f\right)\right]\cdot\frac{1}{2}\left(R_2^f + R_2^e\right) R_2^f$$
$$\frac{\partial\mathcal{O}}{\partial s_1^e} = u''\left(c_1^f\right) + \beta\left[u''\left(\tilde{c}_2^f\right) CE'\left(\tilde{c}_2^f\right)^2 + u'\left(\tilde{c}_2^f\right) CE''\left(\tilde{c}_2^f\right)\right]\cdot\frac{1}{2}\left(R_2^f + R_2^e\right) R_2^e$$

Additional reference

Carroll, Christopher D. and Kimball, Miles S. Liquidity constraints and precautionary saving. NBER Working Paper 8496, 2005.
